# Adolescent alcohol exposure epigenetically regulates CREB signaling in the adult amygdala

**DOI:** 10.1038/s41598-018-28415-9

**Published:** 2018-07-10

**Authors:** Huaibo Zhang, Evan J. Kyzar, John Peyton Bohnsack, Dadasaheb M. Kokare, Tara Teppen, Subhash C. Pandey

**Affiliations:** 1 Center for Alcohol Research in Epigenetics, Department of Psychiatry, University of Illinois at Chicago, Chicago, Illinois 60612 USA; 2 Jesse Brown Veterans Affairs Medical Center, Chicago, Illinois 60612 USA; 3 Department of Anatomy and Cell Biology, University of Illinois at Chicago, Chicago, Illinois 60612 USA

## Abstract

Binge alcohol drinking in adolescence leads to increased risk for alcohol use and other psychiatric disorders in adulthood. The transcription factor cAMP-response element binding (CREB) protein is involved in the neuronal response to adult ethanol exposure, but its role in the enduring effects of adolescent alcohol exposure in adulthood is unknown. We exposed male rats to adolescent intermittent ethanol (AIE) or saline (AIS) during post-natal days 28–41 and evaluated the epigenetic regulation of CREB dynamics in the adult amygdala. A subset of these adult rats was exposed to an acute ethanol challenge. AIE decreased CREB, phosphorylated CREB, CREB-binding protein (CBP) and p300 protein levels in adult amygdaloid brain structures. AIE exposure also causes deficits in *Creb1*, *Cbp*, and *p300* mRNA expression in the amygdala of AIE adult rats which are normalized after acute ethanol exposure. Interestingly, occupancy of acetylated histone H3K9/14 proteins at specific locations in the *Creb1*, *Cbp*, and *p300* gene promoter regions was decreased in the amygdala of AIE adult rats and was normalized by acute ethanol exposure. These results suggest that AIE exposure epigenetically reduces CREB and other related transcriptional activators in the amygdala in adulthood that may be associated with the behavioral effects of adolescent alcohol exposure.

## Introduction

Frequent binge drinking is a risk factor for the development of alcoholism, as ethanol consumption is more likely to result in later addiction if binge amounts are consumed early in life^1,2^. Binge drinking is associated with acute pathophysiological symptoms including learning and memory impairments and disturbances in sleep-wake patterns^3,4^. Long-term sequelae of adolescent binge drinking include increased alcohol use, decreased educational and economic attainment, and increased risk for anxiety and depression^5–7^. Animal models of binge ethanol exposure in adolescence recapitulate many of these phenotypes including increased alcohol consumption and anxiety in adulthood^8–10^. Repeated high-dose alcohol exposure in adolescence leads to neuroadaptations in specific brain circuits and signaling pathways that underlie behavioral changes seen in addiction^11,12^. The limbic system, and particularly the amygdala, plays a critical role in the neurobiology of alcoholism and the negative affective states, including anxiety, that are associated with alcohol abuse^11,13,14^. In humans, the amygdala undergoes a number of structural and connectivity changes during adolescent development^15^, and this process is disrupted by binge alcohol abuse in adolescence^16^. Furthermore, previous studies in rodent models of adolescent binge drinking have indicated that there are substantial changes in signaling pathways in the amygdala that may mediate the increased anxiety-like and alcohol drinking behaviors seen in adulthood in these models^8,9^.

The cAMP-response element binding protein (CREB) is an important transcription factor that is activated by G-protein-coupled receptor signaling cascades and intracellular calcium influx^17^. CREB signaling plays a critical role in neuronal activity and is dysregulated in pathological states such as addiction^18,19^. Neuronal activity causes an increase in CREB phosphorylation^17,18,20^, which leads to nuclear localization and recruitment of CREB binding protein (CBP), p300, and other transcription factors to the chromatin^21,22^. CBP and p300 possess intrinsic histone acetyltransferase (HAT) activity, catalyzing the addition of acetyl groups to N-terminal tails of histone^21–23^. This epigenetic modification is generally associated with increased gene transcription^23^.

Acute ethanol exposure in adult rats increases CREB phosphorylation in the amygdala, while withdrawal after chronic ethanol treatment decreases CREB and CBP expression^24^. Reduced levels of CREB and phosphorylated CREB (pCREB) have been shown in the central nucleus of the amygdala (CeA) and medial nucleus of the amygdala (MeA) of alcohol-preferring (P) adult rats, which display heightened anxiety-like behavior and increased alcohol consumption as compared with alcohol non-preferring (NP) rats^25^. The involvement of CREB, CBP, and p300 in alcohol exposure is not surprising given the intrinsic HAT activity of CBP and p300 and the widespread modulation of epigenetic mechanisms by alcohol^26,27^. In addition, HATs dynamically interact with histone deacetylases (HDACs) to epigenetically regulate synaptic plasticity^28^. We recently demonstrated that a specific HDAC isoform, HDAC2, is increased and histone H3K9 acetylation is decreased in the CeA and MeA after AIE in adulthood^9^. Recently, we also observed that an acute ethanol challenge in adulthood attenuates AIE-induced anxiety-like behaviors^8^. However, the changes in the CREB signaling in adulthood, including the HATs CBP and p300 and their epigenetic regulation, following adolescent alcohol exposure is currently unknown. Therefore, the present study explored CREB signaling and the epigenetic regulation of CREB-related molecules in the amygdala of rats after AIE in adulthood. We also examined the effects of an acute challenge of ethanol on the molecular manifestations of AIE in adulthood in the amygdala. Our results show for the first time that adolescent alcohol exposure causes lasting, epigenetically-encoded deficits in CREB signaling in the amygdala at adulthood.

## Results

### AIE decreases CREB and pCREB levels in the amygdala in adulthood

**Figure 1:**
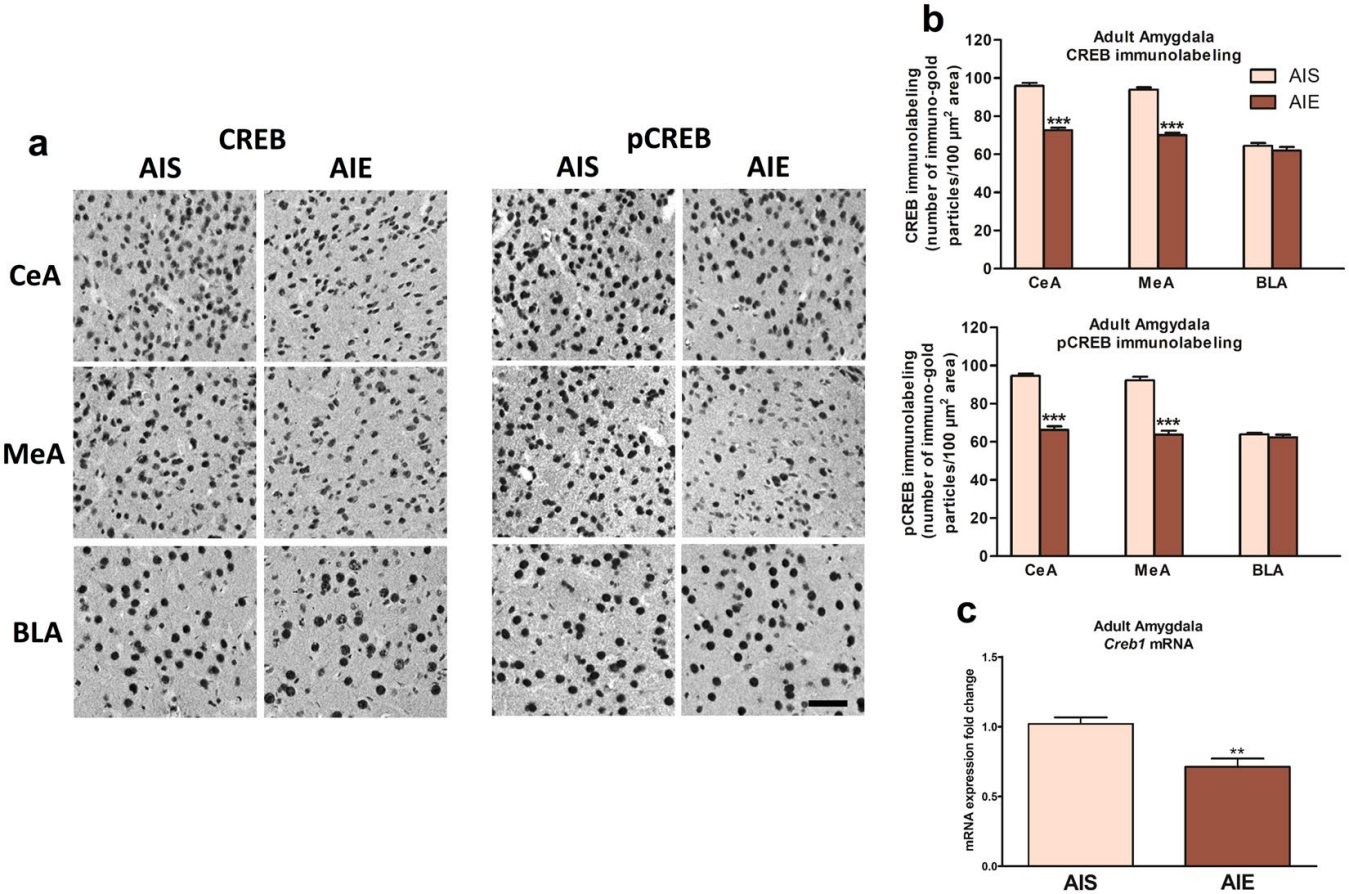
Effects of adolescent intermittent ethanol (AIE) exposure on CREB and pCREB protein levels and *Creb1* mRNA levels in the amygdala of adult rats. (**a**) Representative photomicrographs (Scale bar = 50 μm) of CREB and pCREB gold immunolabeling in the amygdaloid brain structures of AIE and adolescent intermittent saline (AIS) exposed adult rats, and (**b**) bar diagram showing quantification of CREB and phosphorylated CREB (pCREB) gold immunolabeling in the central (CeA), medial (MeA) and basolateral amygdala (BLA) of AIE and AIS adult rats. Values are presented as the mean ± SEM of the number of immuno-gold particles/100 μm^2^. (**c**) Bar diagram showing fold changes in mRNA levels of *Creb1* in the amygdala of AIS and AIE adult rats. Values are presented as the mean±SEM of the fold changes derived from AIS control rats. **p < 0.01, ***p < 0.001, Student’s unpaired two-tailed t-test, n = 6/group (Gold immunolabeling), n = 6/group (mRNA studies).

To explore the lasting influence of adolescent alcohol exposure on CREB signaling-related protein levels, we measured CREB and pCREB levels in the amygdala of AIE and AIS animals in adulthood. We observed a significant reduction in CREB and pCREB protein levels in the CeA (CREB - *t* (10) = 12.26, *p* < 0.001; pCREB - *t*(10) = 12.52, *p* < 0.001) and MeA (CREB - *t*(10) = 13.17, *p* < 0.001; pCREB - *t*(10) = 10.28, *p* < 0.001), but not in the BLA in AIE adult rats as compared to AIS adult rats (Fig. 1a,b). Additionally, *Creb1* mRNA levels are significantly decreased (*t* (10) = 4.15, *p* = 0.002) in the amygdala of AIE adult rats compared to AIS adult rats (Fig. 1c).

### AIE decreases the histone acetyltransferases (HATs) CBP and p300 in the amygdala in adulthood

**Figure 2:**
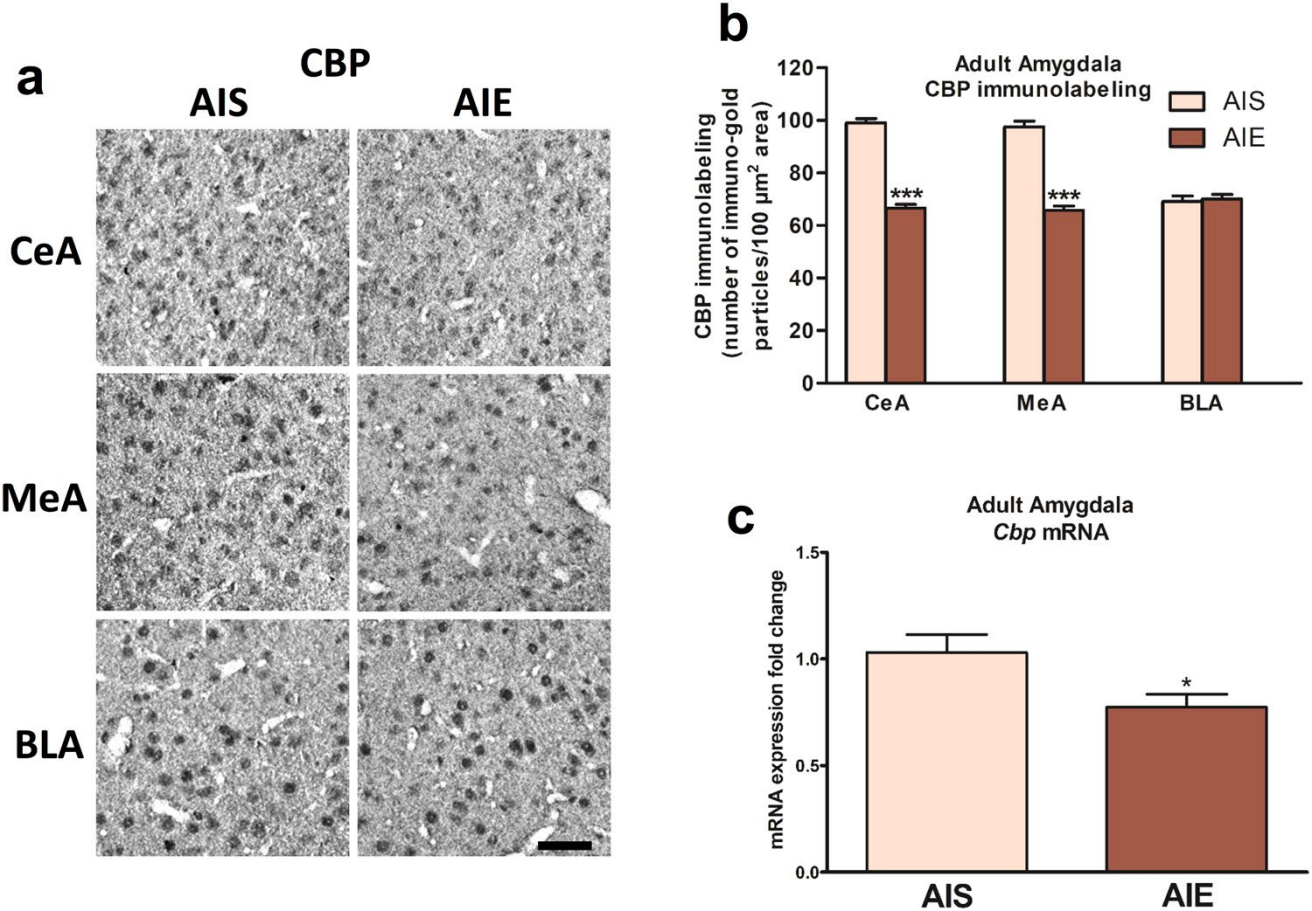
Effects of adolescent intermittent ethanol (AIE) exposure on CREB binding protein (CBP) levels in the amygdala of adult rats. (**a**) Representative photomicrographs (Scale bar = 50 μm) of CBP immunolabeling in the amygdaloid brain structures of AIE and adolescent intermittent saline (AIS) exposed adult rats, and (**b**) Bar diagram showing quantification of CBP gold immunolabeling in the central (CeA), medial (MeA) and basolateral amygdala (BLA) of AIE and AIS adult rats. Values are presented as the mean±SEM of the number of immuno-gold particles/100 μm^2^. (**c**) Bar diagram showing fold changes in mRNA levels of *Cbp* in the amygdala of AIS and AIE adult rats. Values are presented as the mean ± SEM of the fold changes derived from AIS control rats. *p < 0.05, ***p < 0.001, Student’s unpaired two-tailed t-test, n = 6/group (Gold immunolabeling), n = 7–8/group (mRNA studies).

pCREB recruits CBP and the related molecule p300 which both act to transfer acetyl groups to histone proteins and drive transcriptional activity^22,29,30^. Due to the effect of AIE on amygdala CREB and pCREB expression, as well as the involvement of histone acetylation mechanisms in the long-lasting effects of AIE^9^, we measured protein levels of HATs (CBP and p300) in the amygdala following AIE. CBP is significantly decreased in the CeA (*t* (10) = 14.83, *p* < 0.001) and MeA (*t* (10) = 11.53, *p* < 0.001), but not the BLA, of AIE adult rats compared to AIS adult rats (Fig. 2a,b). Additionally, *Cbp* mRNA levels are decreased (*t* (13) = 2.41, *p* = 0.032) in the amygdala of AIE adult rats as compared with AIS adult rats (Fig. 2c).

**Figure 3:**
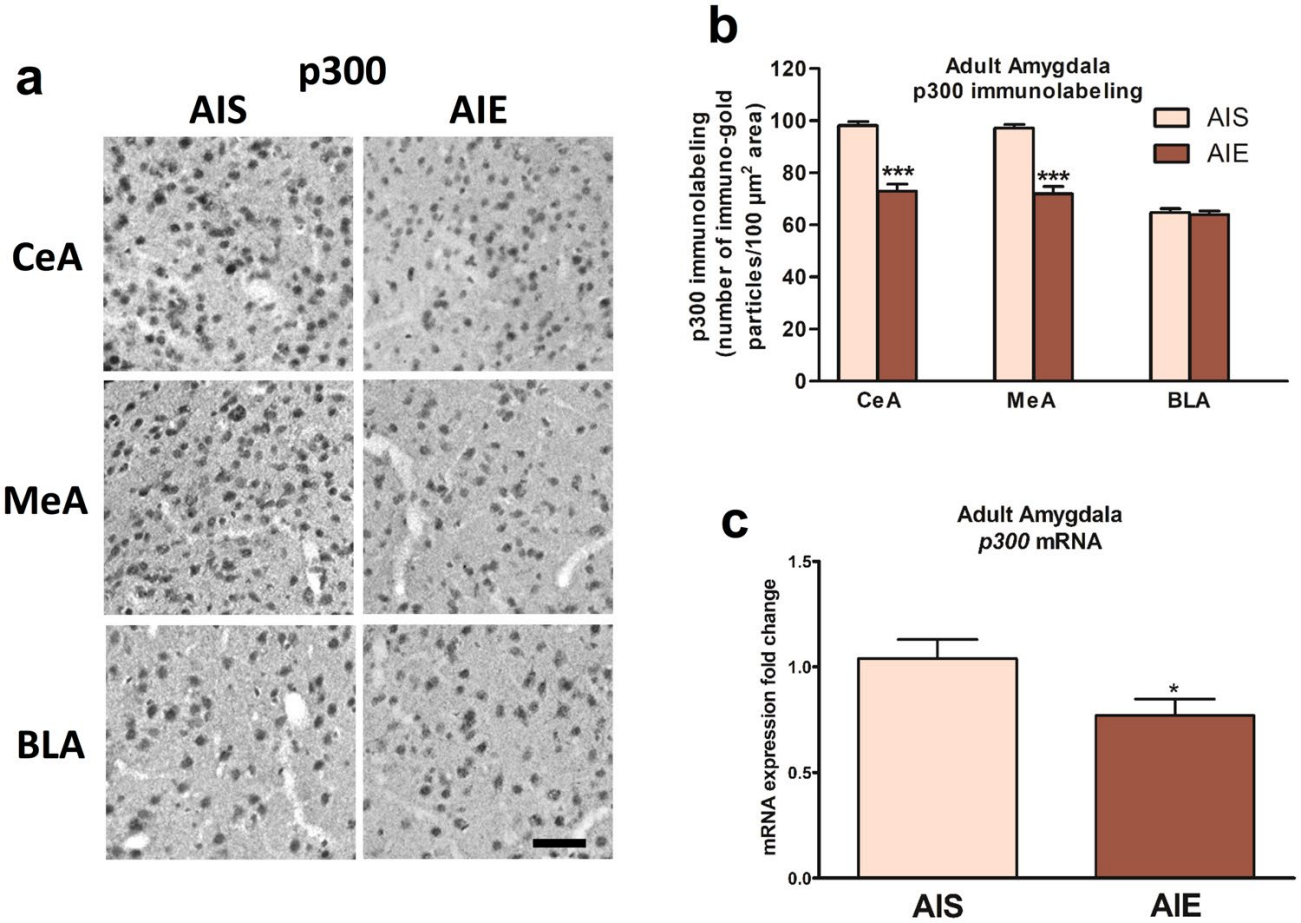
Effects of adolescent intermittent ethanol (AIE) exposure on p300 levels in the amygdala of adult rats. (**a**) Representative photomicrographs (Scale bar = 50 μm) of p300 immunolabeling in the amygdaloid brain structures of AIE and adolescent intermittent saline (AIS) exposed adult rats, and (**b**) Bar diagram showing quantification of p300 gold immunolabeling in the central (CeA), medial (MeA) and basolateral amygdala (BLA) of AIE and AIS adult rats. Values are presented as the mean ± SEM of the number of immuno-gold particles/100 μm^2^. (**c**) Bar diagram showing fold changes in mRNA levels of *p300* in the amygdala of AIS and AIE adult rats. Values are presented as the mean±SEM of the fold changes relative to AIS control rats. *p < 0.05, ***p < 0.001, Student’s unpaired two-tailed t-test, n = 6/group (Gold immunolabeling), n = 7–8/group (mRNA studies).

We next measured p300 protein and mRNA levels in the amygdala of these rats. p300 protein levels are significantly decreased in the CeA (*t* (10) = 8.17, *p* < 0.001) and MeA (*t* (10) = 8.05, *p* < 0.001) of AIE adult rats when compared to AIS adult rats, and there was no significant difference in p300 protein levels in the BLA between groups (Fig. 3a,b). *p300* mRNA levels are also decreased (*t* (13) = 2.19, *p* = 0.047) in the amygdala of AIE adult rats when compared to AIS adult rats (Fig. 3c).

### Acute alcohol challenge in adulthood normalizes AIE-induced changes in mRNA expression in the amygdala

**Figure 4:**
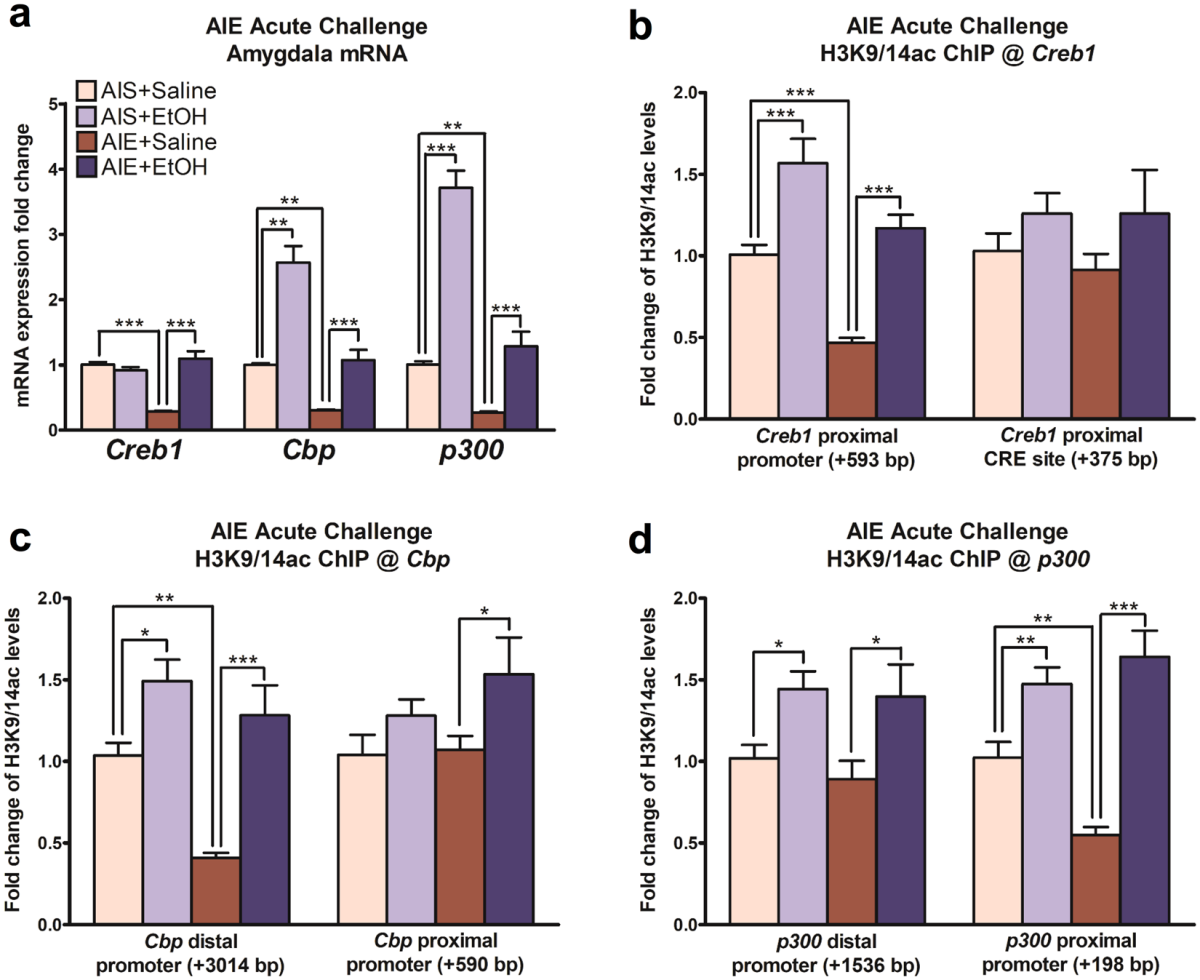
Acute alcohol challenge in adulthood epigenetically normalizes *Creb1*, *Cbp*, and *p300* mRNA expression in the amygdala of rats exposed to adolescent intermittent ethanol (AIE). (**a**) Bar diagram showing fold changes in mRNA levels of *Creb1*, *Cbp*, and *p300* in the amygdala of adolescent intermittent saline (AIS) and AIE rats exposed to an acute challenge of ethanol or saline in adulthood. Occupancy of acetylated histone 3 lysine 9 and 14 (H3K9/14ac) protein was investigated using the chromatin immunoprecipitation (ChIP) assay and primers specific for two sites in the promoter regions of *Creb1* (**b**) *Cbp* (**c**) and *p300* (**d**) in the amygdala of AIS and AIE adult rats exposed to an acute challenge of ethanol or saline. Values are presented as the mean ± SEM of the fold changes relative to AIS + Saline control rats. *p < 0.05, **p < 0.01, ***p < 0.001, two-way ANOVA followed by Tukey’s *post hoc* test, (n = 8–9/group for mRNA studies and n = 5–6/group for ChIP assay). CRE = cAMP response element; bp = base pairs.

We exposed adult rats that had been previously treated with AIE or AIS in adolescence to an acute challenge of alcohol (2 g/kg). Previously, this acute alcohol challenge was shown to normalize the anxiety-like behaviors and transcriptional and epigenetic changes seen in the amygdala of AIE adult rats^10^. We found that there was an overall group effect of AIE on mRNA levels of *Creb1* (*F*(*AIE*)_1,30_ = 15.7, *p* < 0.001) among the groups. Also, significant effects of both acute ethanol exposure (*F*(*acute EtOH*)_1,30_ = 28.1, *p* < 0.001) and the interaction between AIE and acute ethanol (*F*(*AIE x acute EtOH*)_1,30_ = 43.5, *p* < 0.001) were observed. *Post hoc* comparison revealed that AIE significantly (*p* < 0.001) decreased *Creb1* mRNA levels in the amygdala of AIE adult rats, which was normalized (*p* < 0.001) to control levels after acute ethanol exposure (Fig. 4a). We also found that *Cbp* mRNA levels were significantly modulated by AIE (*F* (*AIE*)_1,30_ = 46.7, *p* < 0.001) and acute ethanol exposure (*F* (*acute EtOH*)_1,30_ = 53.0, *p* < 0.001), with a significant interaction of AIE with acute ethanol exposure (*F* (*AIE x acute EtOH*)_1,30_ = 6.20, *p* = 0.019). *Post hoc* comparison indicated a significant reduction (*p* = 0.006) in *Cbp* mRNA levels in the amygdala of AIE adult rats as compared with AIS adult rats. Interestingly, *Cbp* mRNA levels were significantly increased in the amygdala by acute alcohol in both the AIS (*p* < 0.001) and AIE (*p* = 0.002) adult rats (Fig. 4a). Similarly, a two-way ANOVA revealed a significant effect of AIE as well as acute ethanol exposure on *p300* mRNA levels (*F* (*AIE*)_1,29_ = 79.3, *p* < 0.001; *F* (*acute EtOH*)_1,29_ = 109.6, *p* < 0.001), and also a significant interaction between AIE and acute ethanol exposure (*F*(*AIE x acute EtOH*)_1,29_ = 22.5, *p* < 0.001) was observed. Furthermore, *post hoc* comparison indicated a significant decrease by AIE (p = 0.007) and an increase in *p300* mRNA levels by acute alcohol exposure in both AIS (*p* < 0.001) and AIE (*p* < 0.001) adult rats (Fig. 4a). These results suggest that AIE produces long-lasting reductions in the mRNA levels of *Creb1*, *Cbp*, and *p300* in the amygdala which are normalized by acute ethanol challenge in adulthood.

### AIE-induced deficits in histone acetylation of Creb1, Cbp, and p300 promoters in the amygdala at adulthood & reversal by acute ethanol challenge

Given the involvement of epigenetic mechanisms in AIE-induced neuroadaptation^10,31^ and our previous work showing decreased global histone acetylation (H3K9ac) in the CeA and MeA of AIE-exposed adults^9^, we investigated the promoter regions of *Creb1*, *Cbp*, and *p300* for the occupancy of acetylated H3K9/14 (H3K9/14ac) using the chromatin immunoprecipitation (ChIP) assay. We found that H3K9/14ac levels were significantly altered by both AIE exposure (*F*(*AIE*)_1,19_ = 25.2, *p* < 0.001) and acute alcohol challenge in adulthood *F*(*acute EtOH*)_1,19_ = 45.6, *p* < 0.001), but not by the interaction between AIE and acute alcohol, in the *Creb1* proximal promoter site (+593 base pairs [bp] upstream of transcription start site), with *post hoc* testing revealing a decrease in the amygdala of AIE animals at baseline (*p* < 0.001) and an increase by acute alcohol in both AIS (*p* < 0.001) and AIE (*p* < 0.001) adult rats (Fig. 4b). However, there were no significant differences between the groups at a separate *Creb1* promoter (+375 bp) site containing a cAMP response element (CRE; Fig. 4b).

In addition, we found that H3K9/14ac levels in the amygdala were significantly altered by both AIE (*F*(*AIE*)_1,20_ = 12.3, *p* = 0.002) and acute ethanol (*F*(*acute EtOH*)_1,20_ = 31.0, *p* < 0.001), but not the interaction between AIE and acute ethanol, at the *Cbp* distal promoter site (+3014 bp), with AIE adult rats showing a reduction (*p* = 0.001) at baseline compared to AIS adult rats, and both AIS (*p* = 0.014) and AIE adult rats (*p* < 0.001) exposed to acute alcohol displaying increased levels of H3K9/14ac compared to their saline-exposed counterparts (Fig. 4c). Additionally, H3K9/14ac levels at the *Cbp* proximal promoter site (+590 bp) were significantly altered by only acute ethanol exposure (*F* (*acute EtOH*)_1,20_ = 5.95, *p* = 0.024). AIE rats exposed to acute ethanol displayed increased H3K9/14ac levels at the *Cbp* promoter site (+590 bp) compared to AIE adult rats exposed to saline (*p* = 0.034; Fig. 4c).

At the *p300* distal promoter site (+1536 bp), we observed a main effect of altered H3K9/14ac occupancy in the amygdala by acute ethanol (*F* (*acute EtOH*)_1,20_ = 12.2, *p* = 0.002), with AIS (*p* = 0.035) and AIE rats (*p* = 0.015) exposed to an acute ethanol challenge displaying increased H3K9/14ac levels at this site compared to saline-exposed AIS and AIE rats, respectively (Fig. 4d). The occupancy of H3K9/14ac was also significantly different between groups at the *p300* proximal promoter (+198 bp) site with main effects of acute ethanol (*F* (*acute EtOH*)_1,20_ = 49.5, *p* < 0.001) and the interaction between AIE and acute ethanol (*F* (*AIE x acute EtOH*)_1,20_ = 8.55, *p* = 0.008), with *post hoc* testing showing a decrease in the amygdala of AIE animals at baseline (*p* = 0.006) and an increase by acute alcohol in both AIS (*p* = 0.009) and AIE (*p* < 0.001) adult rats (Fig. 4d). These epigenetic changes in the amygdala possibly explain some of the transcriptional alterations in *Creb1*, *Cbp*, and *p300* mRNA produced by AIE in adulthood.

## Discussion

In the present study, we found that AIE produced long-lasting deficits in CREB signaling in the amygdala via histone acetylation mechanisms. The CREB-related signaling factors CBP and p300, possessing intrinsic HAT activity, displayed a decrease in mRNA and protein levels in the CeA and MeA in the adult amygdala after AIE. The deficits in *Creb1*, *Cbp*, and *p300* mRNA levels were normalized in the amygdala of AIE adult rats exposed to an acute challenge of ethanol. We further examined the epigenetic regulation of *Creb1*, *Cbp*, and *p300* mRNA transcription and found a decrease in H3K9/14ac at specific sites in each promoter region in the amygdala of AIE adult rats. Promoter H3K9/14ac levels of these genes returned to normal levels in AIE adult rats exposed to an acute alcohol challenge, mirroring the mRNA expression. Interestingly, changes in H3K9/14ac caused by AIE (and acute ethanol challenge) were specific to certain regions of the promoters, suggesting that localized changes in H3K9/14ac are involved in gene expression changes and are uniquely targeted to genomic regions. Taken together, the decreased CREB signaling seen here may contribute to the increased anxiety-like and alcohol-drinking behaviors seen after AIE in adulthood in these models^8,9^.

The results of this study are consistent with previous reports on the involvement of CREB signaling in alcohol preference and exposure in selectively bred adult rats^25^. Earlier studies show decreased levels of CREB and pCREB, as well as CRE-DNA binding activity, in the amygdala of P rats compared to NP rats^32^. In addition, deficits in the levels of CREB and pCREB were found in amygdaloid structures of P adult rats compared to NP adult rats, specifically in the CeA and MeA, but not the BLA, which correlates with anxiety-like behaviors and higher ethanol consumption in P rats^25^. Treatment with acute ethanol increased levels of pCREB and produced anxiolytic effects in P rats and in mice, but not in NP rats^25,33^. Upstream modulation of the CREB signaling pathway by infusion of a PKA activator (Sp-cAMP) into the CeA increased pCREB levels and decreased anxiety-like behaviors and ethanol consumption of adult P rats. In contrast, infusion of a PKA inhibitor (Rp-cAMP) into the CeA decreased pCREB levels, provoked anxiety-like behaviors, and increased ethanol intake of adult NP rats^25^. Similarly, CREB haploinsufficient adult mice display increased alcohol drinking and anxiety-like behaviors compared to wild-type littermates^33^. Sprague-Dawley adult rats display decreased pCREB levels in the CeA and MeA and increased anxiety-like behavior during withdrawal after chronic ethanol exposure^34^. Sprague-Dawley adult rats consume less alcohol as compared to adult P rats, suggesting that these changes can occur regardless of genetic manipulation and further suggesting that both epigenetic changes and environmental influences such as age of first alcohol exposure contribute to the development of alcohol use disorders.

pCREB activates the downstream signaling molecules CBP and p300, which possess HAT activity^29,30^. Previously, we reported reduced CBP levels in the CeA and MeA and increased anxiety-like behavior in adult rats during ethanol withdrawal after chronic exposure^24^. Other studies have shown that *p300* conditional knock-out mice exhibited reduced histone H3 acetylation in the perirhinal cortex^35^. The decrease in CBP and p300 in the present study corresponds with the decreased global H3K9 acetylation and increased HDAC2 expression reported in the CeA and MeA of AIE adult rats^9^. We have also reported reduced levels of acetylated H3K9/14ac at the promoters of brain-derived neurotrophic factor (*Bdnf*) and activity regulated cytoskeleton-associated protein (*Arc*), genes crucial for synaptic plasticity, and decreased dendritic spine density in the CeA and MeA^9^. Taken together, these results suggest that dynamic interactions between decreased HATs and increased HDACs lead to a condensed chromatin conformation and aberrant synaptic plasticity in the amygdala at adulthood following adolescent alcohol exposure that may be involved in adult psychopathology.

In a previous study, we showed that exposure to an acute challenge of ethanol (2 g/kg) in adulthood normalized AIE-induced anxiety-like behaviors^8^. Here, we show that acute ethanol challenge also rescues the mRNA expression deficits of *Creb1*, *Cbp*, and *p300* in the amygdala. This is mirrored by a decrease in activating H3K9/14ac marks at specific promoter sites of these genes in the amygdala of AIE adult rats that returns to control levels after an acute ethanol challenge. Based on these data we postulate that the long-lasting AIE-induced neuroadaptations in the amygdala contribute to the phenotypes of increased anxiety and alcohol consumption^9^, and that acute exposure to ethanol normalizes these neuroadaptations by increasing H3K9/14ac. This opens the chromatin and allows for increased RNA polymerase binding and subsequent increased transcription of *Creb1*, *Cbp* and *p300*. In AIS-treated animals, we show increases in H3K9/14ac which is consistent with previous studies^24,36^ that demonstrate that acute ethanol exposure in ethanol naïve adult animals leads to open chromatin in the amygdala via increases in CBP and increased global H3K9ac. Our study expands on this previous data and shows that *p300* and *Cbp* are increased after acute ethanol injection in AIS animals due to increases in H3K9/14ac levels in their promoters, suggesting epigenetic regulation of these signaling molecules. Further, the current study suggests that that acute ethanol after AIE increases H3K9/14ac at the promoter regions of *Cbp*, *Creb1* and *p300* which rescues decreases in H3K9/14ac and decreased mRNA levels produced by AIE in adulthood. This is consistent with the rescue of anxiety-like behavior after an acute ethanol injection in AIE adult rats^8^. These findings are particularly interesting in the context of the theory that affective states such as anxiety drive increased alcohol consumption, suggesting the possibility that AIE rats may consume alcohol to restore *Cbp*, *Creb1* and *p300* levels in order to prevent the negative affective states^13,14^. As mentioned above, the decrease in histone acetylation may result from globally increased HDACs^9^ and decreased HATs as observed here in the amygdala in adulthood after AIE. Our results raise the possibility that epigenetic enzymes such as HDAC2 and CBP/p300 change the chromatin conformation around many genes including the gene promoters of epigenetic modifiers themselves, adding multiple layers of complex transcriptional regulation. Additionally, CBP and p300 are known regulators of genomic entities such as enhancers^37,38^, and their global alterations by AIE in specific brain circuits may cause numerous downstream effects that warrant further investigation.

In conclusion, AIE causes lasting changes to the CREB signaling pathway in the amygdala, including decreased CREB, pCREB, CBP and p300 protein levels. This is paralleled by decreased *Creb1*, *Cbp*, and *p300* mRNA in the amygdala that returns to control like levels after an acute challenge of ethanol in adulthood, likely via alterations in H3K9/14ac levels at the respective promoter regions. Collectively, the decreased levels of signaling molecules in the critical CREB pathway seen in this study, along with the increased anxiety and alcohol preference previously reported in AIE adult animals^9^, add to the growing body of evidence that CREB/pCREB/CBP/p300 levels in the amygdala are negatively correlated with anxiety-like and alcohol-drinking behaviors. These novel results suggest that adolescent alcohol causes lasting modifications to the epigenetic dynamics controlling the expression of CREB and related signaling molecules in brain regions crucial for emotion and anxiety. These results may lead to the development of new interventions targeting the epigenome of the alcohol-drinking population who begin alcohol consumption in adolescence.

## Methods

### Animals and adolescent intermittent ethanol (AIE) exposure

Sprague-Dawley (SD) dams with pups or timed-pregnant female rats were purchased from Harlan Laboratories (Indianapolis, IN, USA) and housed under a 12:12 h light/dark cycle with *ad libitum* access to drinking water and food. All experimental protocols strictly adhered to the NIH guidelines for the Care and Use of Laboratory Animals and were approved by the University of Illinois at Chicago Animal Care and Use Committee. Male pups were weaned at post-natal day (PND) 21 and were group-housed (2–3 rats/cage) with *ad libitum* access to water and food maintained. Rats were randomly assigned to receive either adolescent intermittent ethanol (AIE) or adolescent intermittent saline (AIS) treatment. Adolescent male rats received one injection of ethanol (2 g/kg, 20% w/v) or volume-matched n-saline per day via intraperitoneal (i.p.) injection for two consecutive days, followed by 2 days without ethanol treatment for a total of 8 injections of ethanol during PND 28–41. Rats matured without further ethanol exposure until PND 92–102. This schedule of ethanol exposure has been used by our lab and other investigators previously^8,9,39,40^.

### Baseline brain tissue collection

On PND 92, AIS and AIE animals were anesthetized (either with 50 mg/kg of i.p. pentobarbital or inhaled isoflurane) and decapitated, and brain tissues (amygdala) were dissected on ice and quickly frozen. Some rats were perfused with normal saline followed by 4% paraformaldehyde (PFA) solution prepared in 0.1 M phosphate buffer (PB; pH 7.4). Brains were isolated and post-fixed overnight in PFA and soaked in graded sucrose solution (10–20–30%) prepared in PB. All brains were frozen and kept at −80 °C until further use for either biochemical studies or immunohistochemistry.

### RNA isolation and Real-Time Quantitative PCR

Total RNA was extracted from amygdalar tissue using the Qiagen miRNAeasy protocol and DNase Kit (Qiagen, Venlo, NED) by homogenizing frozen tissue on ice using phenol, guanidine isothiocyanate and chloroform. Samples were then treated with RNase-free DNase, and RNA was eluted in RNase-free water. RNA was reverse transcribed using the GeneAmp RNA PCR Core Kit (Life Technologies). Aliquots from each cDNA were amplified by Real-Time PCR using either a Mx3000P qPCR system (Agilent Technologies, Santa Clara, CA, USA) and SYBR Green master mix (Fermentas, Glen Burnie, MD, USA) or a CFX Connect qPCR system with iQ SYBR SuperMix (BioRad, Hercules, CA, USA). Gene of interest expression for *Creb1*, *Cbp*, and *p300* mRNA was examined and glyceraldehyde-3-phosphate dehydrogenase (*Gapdh*) or hypoxanthine-guanine phosphoribosyltransferase 1 (*Hprt1*) mRNA was used as a reference gene. PCR conditions for all primers were 30 s at 95 °C, 30 s at 58 °C and 30 s 72 °C for 40 cycles. Primer sequences are located in Table 1. mRNA data analysis was performed by subtracting the average Ct (crossing threshold) of the reference gene from the gene of interest Ct for each sample. Relative expression levels were then determined using the 2^−ΔΔc(t)^ method to acquire individual fold change^41^. Data is presented as average fold change relative to AIS control animals.

**Table 1:** Primers sets used in this study for mRNA analysis and promoter analysis after chromatin immunoprecipitation.

Primer name	Sequence (5′ to 3′)
**Primers for mRNA analysis via RT-qPCR**	
*Creb1* mRNA forward	AGAAGCAGCACGAAAGAGAG
*Creb1* mRNA reverse	CACTGCCACTCTGTTCTCTAAA
*Cbp* mRNA forward	TAATGGAGGCTGCCCAGTGTGTAA
*Cbp* mRNA reverse	CTGGCGGAGCTTGTGTTTGATGTT
*p300* mRNA forward	AAACACCAGCAACGAGAGTACCGA
*p300* mRNA reverse	TCCATGGTGGCGTACAGTTTCTGA
*Hprt1* mRNA forward	TCCTCAGACCGCTTTTCCCGC
*Hprt1* mRNA reverse	TCATCATCACTAATCACGACGCTGG
*Gadph* mRNA forward	ACAAGATGGTGAAGGTCGGTGTGA
*Gadph* mRNA reverse	AGCTTCCCATTCTCAGCCTTGACT
**Primers for Chromatin Immunoprecipitation**	
*Creb1* proximal promoter (+593) forward	AGTGTCTTGTACTCTGCCGTG
*Creb1* proximal promoter (+593) reverse	ACATAGTGGGGCACAGAGGT
*Creb1* promoter CRE site (+375) forward	GGGATCTGAAGCCAGAATCTCA
*Creb1* promoter CRE site (+375) reverse	TGCACCAGTCAGGTTCAGAAA
*Cbp* distal promoter (+3014) forward	AAAGCTAGCAAGGCGGTAAG
*Cbp* distal promoter (+3014) reverse	CCCTTCCAAAGTGTACGGTAAG
*Cbp* proximal promoter (+590) forward	TCTAGGTCCTGTGCAGCCAT
*Cbp* proximal promoter (+590) reverse	CGGTAGAATTCCTCGTGCTGA
*p300* distal promoter (+1536) forward	AGTGTCCACCGACCGAAAAT
*p300* distal promoter (+1536) reverse	GTCATTCGGTGGCTCCCTTT
*p300* proximal promoter (+198) forward	TCAGTGTTGCTGTACCCTCC
*p300* proximal promoter (+198) reverse	TGCGGACTCAACAGAAATGGT

### Immunohistochemistry

Gold immunolabeling was performed as described previously by our laboratory^24,42^. Coronal brain sections (20 μm) were cut and washed in phosphate buffered saline (PBS), and incubated in RPMI 1640 medium containing l-glutamine (Invitrogen, Carlsbad, CA, USA) for 30 min. Sections were incubated with 10% normal goat serum (NGS) diluted in PBS containing 0.25% Triton X-100 (PBST) for 30 min at room temperature. After blocking the sections with 1% bovine serum albumin (BSA) in PBST, they were incubated with antibodies against CREB (1:500 dilution; Millipore, Billerica, MA, USA, catalog number 06-863), pCREB (1:500; Millipore 06-519), CBP (1:200; Santa Cruz Biotechnology, Dallas, TX, USA, catalog number sc-583) or p300 (1:200; Santa Cruz sc-585) diluted in 1% BSA prepared in PBST for 18 hrs at room temperature. Sections were then washed with PBS followed by 1% BSA in PBS and incubation with gold particle (1.4 nm)-conjugated anti-rabbit secondary antibody (1:200 dilution in 1% BSA in PBS; Nanoprobes, Inc., Yaphank, NY, USA) for 1 hr at room temperature. Gold particles were silver enhanced (Ted Pella Inc., Redding, CA, USA) for 12 to 20 minutes and washed. Quantification of the immunolabeled gold particles was performed by the computerized Image Analyzer Software (Loats Associates, Westminster, MD, USA). Multiple brain sections were taken for each animal in order to assure bregma-matching. A total of nine object fields from 3 amygdala sections from distinct slices were counted for each animal and each amygdaloid structure (CeA, MeA, BLA). Results are represented as the number of immuno-gold particles per 100 μm^2^ area.

### Acute ethanol challenge in adulthood

A subset of AIS and AIE animals were raised and exposed to adolescent alcohol or saline exposure as described above. Additionally, these animals were exposed to an acute challenge either ethanol (2 g/kg; i.p.) or volume-matched n-saline at PND 101-102 to generate 4 total groups (AIS+ Saline, AIS+ EtOH, AIE+ Saline, & AIE+ EtOH) as described previously^8^. One hr after acute ethanol or saline challenge, rats were given anesthesia then sacrificed for the dissection of the amygdala to be used for mRNA analysis (as described above) or chromatin immunoprecipitation (ChIP) assay.

### Chromatin immunoprecipitation (ChIP) assay

The amygdala of animals subjected to acute challenge were also evaluated for acetylated histone 3 lysine 9 and 14 (H3K9/14ac) occupancy at the promoter regions of *Creb1*, *Cbp*, and *p300* using ChIP, as described previously^8,9^. Amygdala tissue was dissected and fixed in 1% methanol-free formaldehyde at 37 °C for 5 min and then quenched with 1 M glycine before homogenization in lysis buffer (1% (v/v) SDS, 10 mM EDTA, 50 mM Tris-HCl pH 8.0). Homogenate was then sonicated using the Covaris ME220 (Covaris, Woburn, MA, USA) to achieve sheared DNA fragments of 200–500 base pairs which was then clarified using centrifugation (17,000 × g for 10 min, 4 °C) to obtain a chromatin fraction. Aliquots of sonicated chromatin were removed as input for normalization in downstream analysis and the remaining sonicated chromatin was incubated with an antibody to H3K9/14ac (Millipore 06–599; 5 µg/sample) overnight at 4 °C. Magnetic Protein A Dynabeads (ThermoFisher Scientific, Waltham, MA, USA) was then added to chromatin samples and rotated for 1 hr 30 min at 4 °C. Chromatin was then washed five times using washing buffer (0.01% SDS, 1.1% Triton X-100, 1.2 mM EDTA, 16.7 mM Tris-HCl pH 8.0, 167 mM NaCl) prior to DNA purification using Chelex-100 resin (10% (w/v), BioRad) by boiling at 95 °C for 10 min. The input aliquot was precipitated using 100% ethanol, washed once with 75% ethanol then purified by using (10% w/v) Chelex-100 resin at 95 °C for 10 min^43^. Purified DNA was analyzed by quantitative PCR using primers directed to specific sites in the *Creb1*, *Cbp*, and *p300* promoter regions (Table 1). The data was analyzed using the ΔΔCt method, normalizing to input, and then data are expressed as fold change relative to AIS+ Saline control rats.

### Statistical analysis

Statistical analysis was conducted using the SigmaStat software suite (Systat Software Inc., San Jose, CA, USA). Differences between adult AIE and AIS groups at baseline were analyzed using Student’s unpaired two-tailed t-test. For experiments involving analysis of acute ethanol challenge, two-way ANOVA (factors: AIE treatment, acute EtOH) followed by Tukey’s *post-hoc* test for multiple comparisons was performed. *p* values of less than 0.05 were considered significant for all tests. Exact *p* values are reported except for cases in which *p* < 0.001. Sample sizes were not pre-calculated but resemble those commonly used in the field and used in previous studies^8,9^. Sample sizes are listed in figure legends as *n* values and are indicative of the number of animals per group in each experiment. All mRNA and ChIP PCR experiments were calculated from technical triplicates or quadruplicates.

### Data availability

All processed data are available in the manuscript. Raw data are available from the corresponding author on reasonable request.
